# Cooking for others is food for the soul: Consistent momentary, but mixed trait‐level well‐being benefits for home cooks

**DOI:** 10.1111/aphw.70121

**Published:** 2026-01-20

**Authors:** Bryant P. H. Hui, Linting Zhang, Jacky C. K. Ng, Johnny C. Y. Lam, Edmond P. H. Choi, Ray Y. H. Cheung, Anise M. S. Wu

**Affiliations:** ^1^ Department of Applied Social Sciences Hong Kong Polytechnic University Hong Kong SAR China; ^2^ Mental Health Research Center Hong Kong Polytechnic University Hong Kong SAR China; ^3^ Department of Social and Behavioral Sciences City University of Hong Kong Hong Kong SAR China; ^4^ School of Nursing University of Hong Kong Hong Kong SAR China; ^5^ Department of Psychology Lingnan University Hong Kong SAR China; ^6^ Department of Psychology, Faculty of Social Sciences University of Macau Macao SAR China; ^7^ Center for Cognitive and Brain Sciences, Institute of Collaborative Innovation University of Macau Macao SAR China

**Keywords:** ecological momentary assessment, prosocial cooking, prosociality, well‐being

## Abstract

Prosocial behavior can promote well‐being, yet the effects of everyday acts—such as cooking for others—remain understudied. Across four studies (*N* > 1,500), we developed and validated a Prosocial Cooking Scale and examined its well‐being effects using cross‐sectional surveys and ecological momentary assessment (EMA). Cross‐sectional analyses linked prosocial cooking to greater positive affect—but also higher negative affect—at the between‐person level. EMA studies revealed within‐person benefits: Individuals reported increased positive affect and subjective happiness—and, in our larger community‐based sample, higher self‐esteem, vitality, and lower negative affect—during prosocial cooking episodes. However, trait‐level associations were modest and inconsistent, emerging most reliably for positive affect. Notably, benefits—including positive affect and self‐esteem—were strongest for introverts, supporting a person‐activity fit perspective. These findings highlight prosocial cooking as an accessible act conferring well‐being gains, and illustrate how EMA captures the impact of kindness in everyday life.

## INTRODUCTION

*A person cooking is a person giving: Even the simplest food is a gift*. —Laurie Colwin, *More Home Cooking* (1993)

Cooking is a universal daily activity with the potential to impact well‐being, yet its psychological benefits for the person preparing food remain understudied. While research has documented that home cooking increased during COVID‐19 pandemic and has remained elevated post‐pandemic (Dezanetti et al., 2022; National Frozen and Refrigerated Foods Association, 2023; Sarda et al., 2022), most studies on cooking and well‐being have focused on specific groups—such as adolescents (Utter et al., 2016) or professional chefs (Ariza‐Montes et al., 2018)—or on therapeutic interventions (Chu et al., 2018; Yu et al., 2023). These investigations tend to emphasize individual outcomes, including socialization, self‐esteem, quality of life, and affect (Farmer et al., 2018; Farmer & Cotter, 2021), without considering cooking as a prosocial behavior or exploring it benefits for the cook.

We propose that cooking for others—what we term “prosocial cooking”—represent an everyday yet overlooked form of prosociality. Prosocial behavior, defined as actions intended to benefit others (e.g., helping, sharing, and providing support), is reliably linked to greater well‐being (Aknin, Barrington‐Leigh, et al., 2013; Curry et al., 2018; Hui et al., 2020). Although preparing food for others is embedded in daily routines and social traditions worldwide (Rossi et al., 2023), psychological science has rarely examined its effects as a distinct form of prosocial behavior. To delineate this construct, we conceptualize prosocial cooking as the intentional preparation of food with the primary aim of benefiting another person. This definition excludes minimal or incidental food‐related actions (e.g., briefly rinsing fruit or handing someone a snack) that require little time, planning, or personal investment. Like many forms of everyday prosocial behavior, however, prosocial cooking does not have perfectly sharp boundaries in real‐world contexts. Rather than constituting a discrete category, it is better understood as a family of related practices that vary in effort, intentionality, and social meaning (Levine et al., 2001). Our definition is therefore intended as a theoretically useful organizing framework rather than a rigid classification, and future research may further refine how different forms and intensities of prosocial cooking relate to well‐being. In the context of sustained shifts toward increased home cooking, understanding whether and how prosocial cooking enhances the well‐being of the cook is both timely and theoretically meaningful.

Although cooking is often viewed as a necessity or recreational pursuit (Szabo & College, 2015), it takes on a social and relational function when directed toward others. Preparing meals can serve as an intentional act of care and generosity, fostering interpersonal connection and appreciation. In this way, prosocial cooking aligns with informal helping—spontaneous, non‐obligatory acts aimed at benefiting others, such as running small errands, holding a door, or giving directions (Hui et al., 2020). Yet it typically involves greater time investment, physical effort, and forethought. These characteristics also distinguish prosocial cooking from other forms of prosocial behavior—such as donating money or volunteering—which lack the same personalized, tangible, and sensory dimensions. At the same time, prosocial cooking remains widely accessible and easily integrated into daily routine, offering a relatively low‐barrier, yet meaningfully effortful way to practice kindness on a regular basis (Rossi et al., 2023). Despite its ubiquity, the construct has rarely been formally defined or systematically investigated for its psychological impact. The present research addresses this gap by conceptualizing prosocial cooking as a unique subtype of prosocial behavior and examining its potential to enhance both momentary and cumulative well‐being for the cook.

To guide our investigation, we draw on the positive‐activity model (Lyubomirsky & Layous, 2013), which posits that intentional positive activities—such as kindness and gratitude—can enhance well‐being through increased positive emotions, stronger social connections, and improved self‐perceptions. In the present research, we use the positive‐activity model primarily to highlight mechanism‐level pathways—such as increases in positive emotion and person‐activity fit—that can operate in real time, rather than to model long‐term intervention effects. The model also highlights that the benefits of such activities depend on factors like frequency, immediacy of rewards, and how well the activity fits individual preferences (person‐activity fit). Prosocial cooking—being a frequent, routine, and socially embedded behavior—is especially likely to generate context‐dependent, short‐term emotional gains. In other words, its psychological rewards are most likely to manifest as immediate, situational boosts in well‐being, rather than as broad, trait‐level differences across individuals. Accordingly, we expected prosocial cooking to show stronger momentary (within‐person) associations with well‐being than stable (between‐person) associations, because the emotional rewards of preparing food for others are likely to be brief and context‐dependent rather than accumulating into enduring trait‐level differences.

To illustrate, consider someone who often cooks for others out of obligation: While they may not report higher overall life satisfaction (a between‐person effect), they may still experience moments of joy, connection, or purpose when preparing meals for loved ones (a within‐person effect). Such context‐dependent benefits are often missed by single‐timepoint or trait‐level assessments. Indeed, research shows that the structure and predictors of well‐being can differ at within‐ and between‐person levels, with meaningful within‐person effects even when between‐person differences are absent (Jayawickreme et al., 2017; Mroczek et al., 2003; Rush & Hofer, 2014). Thus, while prosocial cooking may not reliably predict higher trait‐level well‐being, it likely provides consistent, emotionally salient benefits in daily life.

## THE PRESENT STUDY

Although originally formulated to account for long‐term changes in well‐being, the positive‐activity model (Lyubomirsky & Layous, 2013) also highlights mechanisms—such as increases in positive emotion—that can unfold in real time. This makes prosocial cooking a useful context for examining short‐term emotional processes. However, many studies on prosocial behavior and well‐being have relied on cross‐sectional, between‐person designs (Hui et al., 2020), which may overlook dynamic, within‐person emotional processes involved in everyday acts like cooking. To address this gap, we first developed and validated a brief, practical measure of prosocial cooking (Studies 1a and 1b), providing a foundation for investigating its well‐being correlates. Although our main hypothesis concerns momentary effects, establishing a robust measure and exploring trait‐level associations were essential groundwork and a benchmark for comparison. In Studies 2 and 3, we used ecological momentary assessment (EMA) to capture real‐time emotional and psychological experiences in daily life, enabling us to rigorously test both within‐person fluctuations and aggregated between‐person effects of prosocial cooking. This approach also provided a direct empirical contrast to the cross‐sectional findings from Study 1b.

We hypothesized that prosocial cooking would be associated with well‐being both at the trait (between‐person) and momentary (within‐person) levels, but expected stronger, more consistent effects at the within‐person level, reflecting its context‐dependent nature. Finally, guided by the positive‐activity model's person‐activity fit (Lyubomirsky & Layous, 2013) and recent calls to address heterogeneity in behavioral science (Bryan et al., 2021), we explored whether personality traits moderate the well‐being benefits of prosocial cooking. Specifically, we examined the Big Five personality traits, which are robustly linked to happiness (Costa & McCrae, 1980; Steel et al., 2008). Although exploratory, extraversion emerged as a particularly relevant trait. Extraverts, who often gain well‐being from frequent, high‐intensity social interactions, might experience smaller incremental benefits from routine prosocial acts like cooking. In contrast, introverts, who tend to prefer more predictable, low‐arousal forms of engagement, may find structured, everyday prosocial activities such as cooking especially well‐suited to their social preferences. This perspective aligns with findings that introverts derive greater benefit from low‐stimulation activities that fit their comfort zones (Zelenski et al., 2013).

## STUDY 1A: DEVELOPING A MEASURE OF PROSOCIAL COOKING

Although cooking for others is a widespread practice, there is currently no validated measure of it. Accordingly, Study 1a aimed to develop the Prosocial Cooking Scale and examine its underlying structure through exploratory factor analysis (EFA).

## METHOD

### Participants and Procedure

Scale items were developed based on the conceptualization of prosocial cooking and a review of existing prosocial behavior measures (e.g., Hui et al., 2020), then iteratively refined by the research team for clarity and content validity. Participants were 503 adults (*M*_age_ = 42.78, *SD*_age_ = 11.79; 56.1% female; See Table 1 for more demographic information) recruited from the Hong Kong community via Kantar, an international research panel provider. A sensitivity power analysis conducted with G*Power indicated that, assuming 90% power and a sample size of 503, the smallest detectable effect size was *r* = .14 (two‐tailed α = .05). After providing consent, participants completed an online survey including the 5‐item Prosocial Cooking Scale among other unrelated measures; only data relevant to this study are reported here. Participants were asked, “In the past week, how many times have you done the following behaviors?” One sample item is “cooking lunch for others (including lunch boxes),” rated on an 8‐point scale (0 = *not at all*, 7 = *seven times or above*) (see Table 2 for all items).

**TABLE 1:** Characteristics of Participants in all Studies.

	Study 1a (*N* = 503)	Study 1b (*N* = 520)	Study 2 (*N* = 231)	Study 3 (*N* = 295)
	Community participants	Community participants	College students	Community participants
Characteristics	*M* (*SD*) or *n* (%)	*M* (*SD*) or *n* (%)	*M* (*SD*) or *n* (%)	*M* (*SD*) or *n* (%)
**Age**	42.78 (11.79)	42.84 (11.52)	18.93 (1.32)	37.31 (12.29)
**Gender** (Female/Male)	Female: 282 (56.1%)	Female: 296 (56.9%)	Female: 144 (62.3%)	Female: 232 (78.6%)
**Race/Ethnicity** (Chinese/Other)	Chinese: 483 (96.0%)	Chinese: 520 (100%)	Chinese: 231 (100%)	Chinese: 295 (100%)
**Education level**				
Primary school	2 (0.4%)	0	‐‐	2 (0.7%)
Secondary school/High school	119 (23.7%)	128 (24.6%)	‐‐	52 (17.6%)
Certificate/Diploma	51 (10.1%)	37 (7.1%)	‐‐	9 (3.1%)
Associate degree/Higher diploma	47 (9.3%)	49 (9.4%)	‐‐	25 (8.5%)
Bachelor's degree	219 (43.5%)	266 (51.2%)	‐‐	146 (49.5%)
Master's degree	62 (12.3%)	40 (7.7%)	‐‐	54 (18.3%)
Doctoral degree	3 (0.6%)	0	‐‐	7 (2.4%)
**Annual Income**				
No income	22 (4.4%)	17 (3.3%)	‐‐	33 (11.2%)
Less than HKD 4,000	16 (3.2%)	19 (3.7%)	‐‐	19 (6.4%)
HKD 4,000‐9,999	27 (5.4%)	13 (2.5%)	‐‐	20 (6.8%)
HKD 10,000‐19,999	105 (20.9%)	94 (18.1%)	‐‐	62 (21.0%)
HKD 20,000‐29,999	136 (27.0%)	143 (27.5%)	‐‐	74 (25.1%)
HKD 30,000‐39,999	86 (17.1%)	104 (20.0%)	‐‐	51 (17.3%)
HKD 40,000‐49,999	41 (8.2%)	34 (6.5%)	‐‐	20 (6.8%)
HKD 50,000‐59,999	19 (3.8%)	50 (9.6%)	‐‐	5 (1.7%)
HKD 60,000‐69,999	17 (3.4%)	28 (5.4%)	‐‐	1 (0.3%)
HKD 70,000‐79,999	13 (2.6%)	5 (1.0%)	‐‐	6 (2.0%)
HKD 80,000 or above	21 (4.2%)	13 (2.5%)	‐‐	4 (1.4%)
**Marital status**				
Single/Never married	209 (41.6%)	173 (33.3%)	‐‐	175 (59.3%)
Married	281 (55.9%)	328 (63.1%)	‐‐	107 (36.3%)
Separated	0	0	‐‐	2 (0.7%)
Divorced	11 (2.2%)	15 (2.9%)	‐‐	9 (3.1%)
Widowed	2 (0.4%)	4 (0.8%)	‐‐	2 (0.7%)

*Note:* The notation “*n* (%)” represents the number of participants (*n*) and the corresponding percentage (%) of the total. No information about education level, annual income, and marital status was collected in Study 2.

Abbreviations: *M* = Mean; *SD* = Standard Deviation.

**TABLE 2:** Standardized Factor Loadings of the Prosocial Cooking Scale in Study 1a.

Items	Loadings
1. Cooking breakfast for others (including making bread)	.85
2. Cooking lunch for others (including lunch boxes)	.90
3. Cooking afternoon tea for others (e.g. muffins, sandwiches, tea)	.83
4. Cooking dinner for someone else	.76
5. Cooking a late‐night snack for someone else	.64

## RESULTS

### EFA

We conducted an EFA in SPSS 22 using principal axis factoring with oblimin rotation. Factor retention was determined jointly by examining eigenvalues, inspecting the scree‐plot, and referencing parallel analysis (Carpenter, 2018). The Kaiser‐Meyer‐Olkin (KMO) measure of sampling adequacy was .85 and Bartlett's test of sphericity was significant (*p* < .001), indicating suitability for factor analysis.

The initial solution revealed a single factor with an eigenvalue greater than 1, explaining 70.48% of the variance. The second factor explained only 12.66% of the variance, representing a substantial drop from the first factor. The scree‐plot also showed a clear decline after the first factor. Parallel analysis indicated that the eigenvalue for the second factor was below the mean of random eigenvalues, further supporting a one‐factor solution. Thus, both factor analytic criteria and parallel analysis supported a single‐factor structure. Item loadings are presented in Table 2. The scale also demonstrated good reliability (ω = .90).

## STUDY 1B: CONFIRMING SCALE STRUCTURE AND ASSOCIATIONS WITH WELL‐BEING

Study 1b aimed to validate the Prosocial Cooking Scale using confirmatory factor analysis (CFA) and to examine its cross‐sectional associations with well‐being, controlling for demographic covariates (age, gender), social desirability, and general prosocial behavior.

## METHOD

### Participants and Procedure

An independent sample of 520 Hong Kong community participants (*M*_age_ = 42.84, *SD*_age_ = 11.52; 56.9% female; More demographic information is presented in Table 1) was recruited through Kantar, distinct from the EFA sample used in Study 1a. A sensitivity power analysis conducted with G*Power showed that, assuming 90% power and a sample size of 520, effects as small as r = .14 could be detected (two‐tailed α = .05). After providing consent, participants completed an online survey that began with measures assessing prosocial cooking and general prosocial behavior, followed by well‐being, social desirability, and relevant covariates. The Measures section below follows the same order.

## MEASURES

### Prosocial Cooking

Prosocial cooking was assessed by the five‐item Prosocial Cooking Scale (see Study 1a).

### General Prosocial Behavior

General prosocial behavior was assessed by the 10‐item Hong Kong Altruism Index (Cheng et al., 2017), which captures informal helping (e.g., offering seats on public transport to a stranger), formal helping (e.g., volunteering), monetary donation, and blood and organ donation. Participants indicated whether they had engaged in each act within a specific period.

### Positive and Negative Affect

The 20‐item Positive and Negative Affect Schedule (PANAS; Watson et al., 1988) was used to assess positive (e.g., interested, excited) and negative (e.g., irritable, distressed) affect in the past week, with items rated on a 5‐point Likert scale (1 = *very slightly or not at all*, 5 = *extremely*).

### Subjective Happiness

Subjective happiness was measured with the four‐item Subjective Happiness Scale (Lyubomirsky & Lepper, 1999), using a 7‐point Likert scale. Sample items include, “Generally, I considered myself: 1 (*not a very happy person*) to 7 (*a very happy person*)” and “Compared to most of my peers, I considered myself: 1 (*less happy*) to 7 (*more happy*).”

### Social Desirability

Because prosocial cooking inherently involves helping or caring for others, participants may respond in a socially desirable manner when reporting such behaviors. Therefore, social desirability was included as a control variable when examining the association between prosocial cooking and well‐being. Social desirability was measured using a six‐item short‐form of the Marlowe‐Crowne Social Desirability Scale (Fischer & Fick, 1993). A sample item is “I have never intensely disliked anyone,” with dichotomous (true/false) response options.

Reliabilities of all scales are shown in Table 3. Prosocial behavior was assessed using subscale and total scores. For descriptive and correlation analyses, both were computed, while only the total score was used in regression analyses to ensure parsimony and avoid multicollinearity. A total score was calculated for social desirability, and the means of all items or subscale items were used for other variables.

**TABLE 3:** Descriptive Statistics and Inter‐correlation Coefficients of Study Variables in Study 1b.

	*M*	*SD*	ω	1	2	3	4	5	6	7	8	9	10
1. Prosocial cooking	1.28	1.51	.88	‐‐									
2. Positive affect	2.81	0.82	.92	.40***	‐‐								
3. Negative affect	2.37	1.02	.95	.24***	.49***	‐‐							
4. Subjective Happiness	4.24	1.22	.83	.15***	.51***	−.19***	‐‐						
5. General prosocial behavior	4.24	2.63	.77	.40***	.49***	.28***	.23***	‐‐					
6. Formal helping	0.63	0.82	.70	.33***	.46***	.39***	.18***	.75***	‐‐				
7. Monetary donation	0.63	0.48	‐‐	.22***	.27***	.08	.19***	.62***	.34***	‐‐			
8. Blood and organ donation	0.69	.74	.43	.10*	0.06	−.10*	.04	.43***	.11**	.21***	‐‐		
9. Informal helping	2.28	1.56	.71	.38***	.48***	.28***	.22***	.90***	.59***	.45***	.13**	‐‐	
10. Social desirability	3.60	1.48	.48	−.04	−.03	−.37***	.25***	−.02	−.07	.001	.10*	−.05	‐‐
11. Gender	‐‐	‐‐	‐‐	−.17***	−.14**	−.18***	−.08	−.20***	−.16***	−.14**	−.03	−.19***	.14**
12. Age	42.84	11.52	‐‐	−.09*	−.30***	−.43***	.10*	−.26***	−.37***	−.04	.09*	−.28***	.20***

*Note:* Variables 6 through 9 are sub‐factors of prosocial behavior; the ω column presents the omega reliability coefficients for each variable. Monetary donation has only one item, so reliability is not applicable. Consistent with previous research on short‐form Marlowe‐Crowne scales—which typically report reliabilities ranging from .35 to .62 (e.g., Beretvas et al., 2002; Tan et al., 2022; Thompson & Phua, 2005)—the scale demonstrated a similar level of reliability in the current sample (ω = .48). For gender, female is coded as 0 and male is coded as 1.

Abbreviations: M = Mean; SD = Standard Deviation.

* *p* < .05,

** *p* < .01,

*** *p* < .001.

## RESULTS

### CFA

**FIGURE 1:**
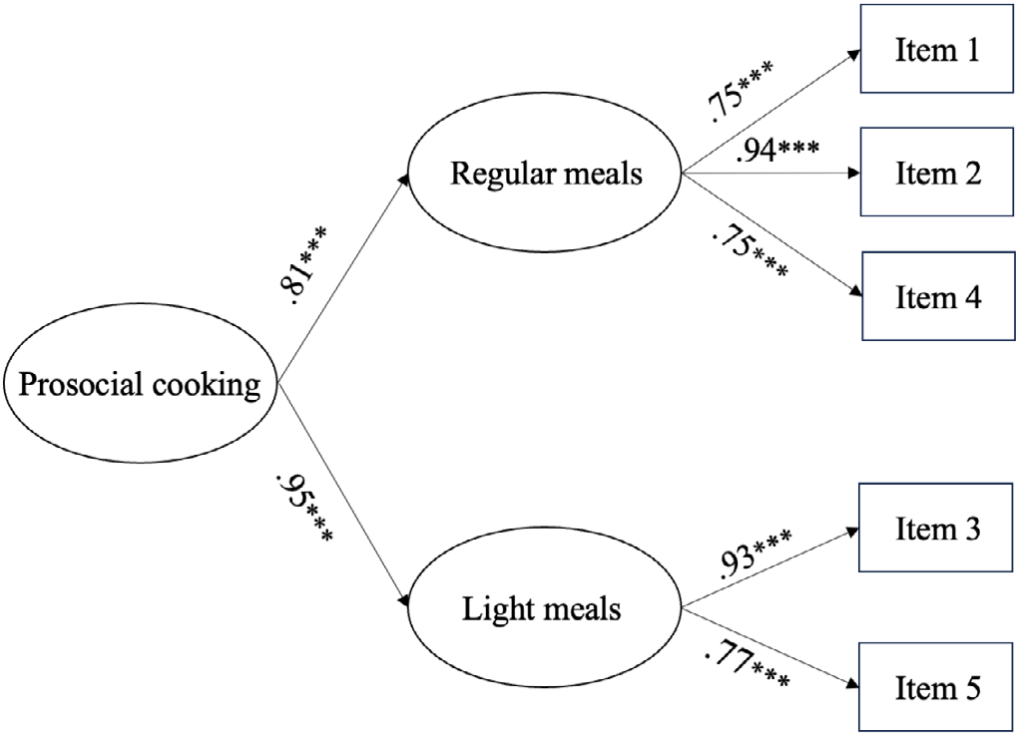
Higher‐order Factor Model of Prosocial Cooking in Study 1a. Note. Standardized factor loadings are presented.

We first conducted a CFA using maximum likelihood estimation with robust standard errors in Mplus to validate the structure of the Prosocial Cooking Scale. The initial single‐factor model, with all five items loaded onto one latent factor, did not fit the data adequately, χ^2^(5) = 76.66, *p* < .001; Comparative Fit Index (CFI) = .88; Tucker‐Lewis Index (TLI) = .76; Root Mean Square Error of Approximation (RMSEA) = .17; Standardized Root Mean Square Residual (SRMR) = .06. Modification indices suggested that correlating the error terms of items 3 (afternoon tea) and 5 (late‐night snack) would substantially improve model fit. Conceptually, both items represented light meals, whereas the other items were regular meals. Accordingly, we specified a higher‐order model, with prosocial cooking captured by two related latent factors (regular and light meals) (see Figure 1 for the model and standardized factor loadings). This model fit the data well, χ^2^(3) = 8.13, *p* = .043; CFI = .99; TLI = .97; RMSEA = .06; SRMR = .02.

### Internal Consistency and Convergent Validity

The Prosocial Cooking Scale demonstrated high internal consistency (ω = .88). For convergent validity, prosocial cooking was positively correlated with general prosocial behavior (*r* = .40, *p* < .001), informal helping (*r* = .38, *p* < .001), formal helping (*r* = .33, *p* < .001), and monetary donation (*r* = .22, *p* < .001), as expected given their conceptual overlap (see Table 3). These findings support the scale's reliability and its convergence with established constructs.

### Cross‐sectional Associations Between Prosocial Cooking and Well‐being

Descriptive statistics and correlations are presented in Table 3. Prosocial cooking was positively correlated with positive affect (*r* = .40, *p* < .001), negative affect (*r* = .24, *p* < .001), and subjective happiness (*r* = .15, *p* < .001). Hierarchical regression analyses were conducted to predict well‐being, with age, gender, and social desirability entered in the first step, general prosocial behavior in the second, and prosocial cooking in the third. As shown in Table 4, after accounting for age, gender, social desirability, and general prosocial behavior, prosocial cooking significantly and positively predicted positive affect (β = .24, 95% CI [.16, .32], *p* < .001, Δ*R* ^*2*^ = .04, *p* < .001) and negative affect (β = .13, 95% CI [.05, .21], *p* = .001, Δ*R* ^*2*^ = .01, *p* = .001), but did not significantly predict subjective happiness (β = .08, 95% CI [−.01, .16], *p* = .095, Δ*R* ^*2*^ = .00, *p* = .095). Thus, at the between‐person level, greater engagement in prosocial cooking was associated with higher positive affect and, unexpectedly, higher negative affect. No association was observed with subjective happiness.

**TABLE 4:** Hierarchical Regression Models for Variables Predicting Well‐being in Study 1b.

Variables	Positive affect			Negative affect			Subjective happiness		
	Block 1, β	Block 2, β	Block 3, β	Block 1, β	Block 2, β	Block 3, β	Block 1, β	Block 2, β	Block 3, β
Gender	−.14**	−.06	−.03	−.13**	−.09*	−.08*	−.11**	−.06	−.06
Age	−.30***	−.19***	−.19***	−.37***	−.32***	−.32***	.05	.12**	.12**
Social desirability	.05	.02	.03	−.28***	−.29***	−.29***	.26***	.25***	.25***
Prosocial behavior		.43***	.34***		.17***	.12**		.26***	.23***
Prosocial cooking			.24***			.13**			.08
*R* ^2^	.11	.28	.32	.28	.31	.32	.08	.14	.14
Δ*R* ^2^	.11	.17	.04	.28	.03	.01	.08	.06	.00
*F*_change_	21.09***	119.93***	35.59***	67.32***	18.83***	11.18**	14.96***	34.63***	2.79
Δ*df*	3/516	1/515	1/514	3/516	1/515	1/514	3/516	1/515	1/514

*Note*: Standardized regression coefficients are presented. For gender, female is coded as 0 and male is coded as 1.

* *p* < .05,

** *p* < .01,

*** *p* < .001.

## STUDY 2: EMA OF PROSOCIAL COOKING AND WELL‐BEING AMONG COLLEGE STUDENTS

While Study 1b provided preliminary evidence for between‐person associations between prosocial cooking and well‐being, its cross‐sectional design was limited in capturing the dynamic, real‐time processes that occur in daily life. To address this limitation, Study 2 employed EMA to examine both momentary, within‐person fluctuations in well‐being and aggregated, between‐person effects derived from repeated assessments across one week (three times per day). Aggregated means from multiple EMA observations provided a more accurate and ecologically valid estimate of individuals' typical experiences than single, retrospective cross‐sectional assessments. Also, Study 2 enabled a direct comparison of within‐person and between‐person associations, leveraging a more robust and context‐sensitive methodology than prior cross‐sectional research.

## METHOD

### Participants and Procedure

A priori power analysis, conducted via Monte Carlo simulation (Arend & Schäfer, 2019) using the *simr* package in R, indicated that a sample of 150 participants with 21 repeated measures would provide 90% power to detect small within‐person effects (γ_std_ = .10, ICC = .50, α = .05). We recruited 233 college students who participated for course credit. To ensure sufficient data for reliable within‐person estimation, we excluded participants with fewer than three completed EMA entries, as this represents less than one full day of sampling (three prompts per day). The final sample comprised 231 college students (*M*_age_ = 18.93, *SD*_age_ = 1.32; 62.3% female; Detailed demographic information is shown in Table 1).

All study procedures—including informed consent, the baseline survey, and EMA prompts—were administered through the Longitudinax Pro mobile app (compatible with iOS/Android). After completing the baseline measures and demographic items (i.e., age and gender) in the app, participants began the 7‐day EMA protocol on the Friday following their baseline survey to ensure consistency and control for potential weekday/weekend effects.

During the EMA week, participants received notifications to complete brief surveys at 11:00 a.m., 4:00 p.m., and 9:00 p.m. daily. Each survey window was open for two hours, and a reminder was sent one hour after the initial prompt if no response had been recorded. The three daily prompts were scheduled to cover most possible meal occasions, thereby maximizing opportunities to capture episodes of prosocial cooking across diverse eating contexts, as suggested by the patterns in Studies 1a and 1b. Within each prompt, participants first completed the momentary prosocial cooking measure, followed by assessments of momentary positive affect, negative affect, and happiness. In total, 3,864 EMA entries were collected, with participants completing an average of 16.73 entries (*SD* = 4.43) out of 21 possible entries over the study week (compliance rate = 79.7%).

## MEASURES

### Self‐oriented Cooking

Self‐oriented cooking was assessed with one item: “In the past week, how many times did you cook for yourself (not including others)?” rated on an 8‐point scale ranging from 0 to 7 times or above. Because the amount of time people spend cooking for themselves likely varies widely across individuals and may be difficult to estimate accurately, we measured the frequency rather than total time as a simpler, potentially more reliable indicator of individual differences in self‐oriented cooking.

### Social Desirability

Social desirability was measured using the same six‐item short‐form Marlowe‐Crowne scale as in Study 1b (ω = .41).

### Momentary Prosocial Cooking

To measure prosocial cooking in daily life, we adapted the core item structure from the Prosocial Cooking Scale developed in Study 1, modifying the response format for EMA. The original scale used “number of times” over a week, which is unsuitable for EMA's short, repeated assessment windows, as participants are unlikely to cook for others multiple times within a few hours—resulting in range restriction and limited variance. Instead, participants reported the time spent preparing food or cooking for others in the past couple of hours, using a categorical scale (0, 0.5, 1, 1.5, 2, 2.5, or 3 hours). This adaptation preserved conceptual alignment with Study 1 while enabling more sensitive, event‐based measurement appropriate for EMA.

### Momentary Positive and Negative Affect

The 9‐item Emmons Mood Indicator (Diener & Emmons, 1985) was used to measure momentary positive (e.g., joyful, happy) and negative affect (e.g., depressed, unhappy). Participants indicated the extent to which they had experienced each emotion in the past couple of hours on a 7‐point Likert scale (1 = *not at all*, 7 = *extremely*).

### Momentary Subjective Happiness

Momentary subjective happiness was assessed with a two‐item version of the Subjective Happiness Scale (Lyubomirsky & Lepper, 1999), modified to reference the past couple of hours (Hui & Kogan, 2018).

Reliabilities for EMA scales are presented in Table 5. Mean subscale scores were calculated for momentary positive and negative affect. Momentary subjective happiness was measured by the mean of all items.

**TABLE 5:** Descriptive Statistics and Inter‐correlations among the Main Study Variables in Study 2.

Variables	*M*	Variance		ICC	ω		1	2	3	4
		Within	Between		Within	Between				
1. Prosocial cooking	0.08	0.09	0.03	.28	‐‐	‐‐	‐‐	.14*	.06	.12
2. Positive affect	4.26	0.72	1.09	.61	.88	.99	.06**	‐‐	−.47***	.84***
3. Negative affect	2.83	0.71	1.03	.59	.80	.97	−.02	−.38***	‐‐	−.58***
4. Subjective happiness	4.38	0.52	1.45	.74	.70	.98	.05	.50***	−.30***	‐‐

*Note*: Descriptive statistics and inter‐correlations are based on the aggregate observed mean for each person. *M* = mean; ICC = intraclass correlation. The ω column presents the omega reliability coefficients for each variable. Prosocial cooking has only one item, so reliability is not applicable. Within‐level correlations are placed on the lower left and between‐level correlations are placed on the upper right.

* *p* < .05;

** *p* < .01;

*** *p* < .001.

## RESULTS

To account for the hierarchical data structure (momentary assessments nested within individuals), we employed multilevel modeling using Mplus 8.3. Standardized effects (β) were reported along with the 95% credibility interval (CI) for all effects.

### Preliminary Analyses

Following Bolger and Laurenceau (2013), we first estimated descriptive statistics, correlations, and intraclass correlation coefficients (ICCs) using null (intercept‐only) models (see Table 5). ICCs for the main study variables ranged from .28 to .74, indicating substantial variance at within‐person and between‐person levels, justifying the use of multilevel modelling.

### Multilevel Analyses

**FIGURE 2:**
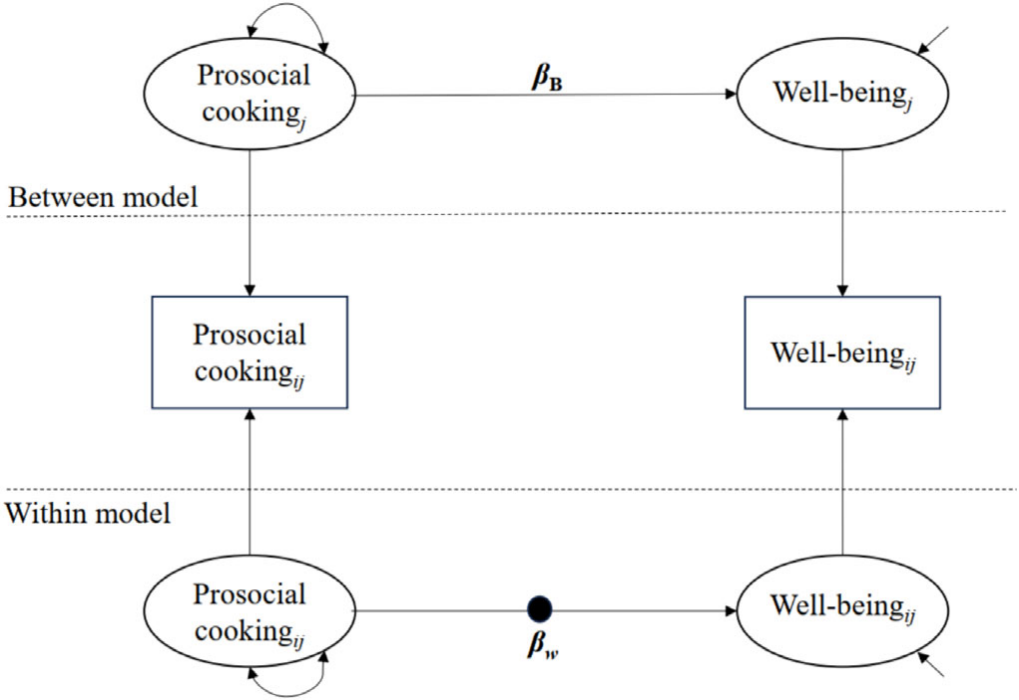
The Multilevel Structural Equation Model Used in Studies 2. Note. The filled‐in circle is random slope. β_B_ represents the coefficient of the between‐person level effect and β_W_ denotes the coefficient of the within‐person level effect.

We then examined within‐ and between‐person associations between prosocial cooking and well‐being by regressing well‐being outcomes (positive affect, negative affect, subjective happiness) on prosocial cooking, with random intercepts and slopes (see Figure 2). Bayesian estimation with default diffuse (noninformative) priors was used. Predictors were latent‐mean‐centered. Age, gender, and social desirability were included as level‐2 covariates.

We found that, at the within‐person level, prosocial cooking significantly predicted greater same‐session positive affect, β = .065, 95% CI [0.023, 0.099], and subjective happiness, β = .050, 95% CI [0.008, 0.089]. The association with negative affect was negative as expected but not statistically significant, β = −.026, 95% CI [−0.064, 0.009]. At the between‐person level, prosocial cooking was not significantly associated with any well‐being outcomes (all|β| < .126, 95% CIs included zero; see Table 6 for details). These findings suggest that engaging in prosocial cooking was reliably linked to enhanced positive affect and happiness in the moment, although the association with reduced negative affect was not statistically robust. There was no evidence that prosocial cooking predicted between‐person differences in well‐being. Variance estimates for the within‐person slopes of prosocial cooking predicting positive affect (0.014, 95% CI [0.001, 0.055]), negative affect (0.033, 95% CI [0.004, 0.108]), and subjective happiness (0.075, 95% CI [0.034, 0.141]) indicated substantial individual differences in these associations. This pattern motivated our examination of personality moderators in Study 3.

**TABLE 6:** Multilevel Models in Studies 2 and 3 with Prosocial Cooking as the Predictor.

	Study 2	Study 3
Outcomes	β	β
Positive affect		
Intercept	3.883 [3.470, 4.292]	3.422 [3.103, 3.800]
Within	.**065 [0.023, 0.099]**	.**078 [0.057, 0.100]**
Between	.126 [−0.016, 0.262]	.**168 [0.042, 0.273]**
Negative affect		
Intercept	2.663 [2.372, 2.965]	2.139 [1.895, 2.388]
Within	−.026 [−0.064, 0.009]	**−.041 [−0.068, −0.016]**
Between	.076 [−0.056, 0.205]	.016 [−0.103, 0.137]
Subjective happiness		
Intercept	3.481 [3.109, 3.863]	3.207 [2.913, 3.546]
Within	.**050 [0.008, 0.089]**	.**061 [0.036, 0.081]**
Between	.109 [−0.028, 0.242]	.089 [−0.036, 0.212]
Self‐esteem		
Intercept	‐‐	5.602 [5.098, 6.134]
Within	‐‐	.**066 [0.042, 0.092]**
Between	‐‐	.013 [−0.103, 0.133]
Vitality		
Intercept	‐‐	2.782 [2.511, 3.085]
Within	‐‐	.**056 [0.036, 0.078]**
Between	‐‐	.103 [−0.016, 0.216]
Pseudo *R* ^2^	.027	.048

*Note*: β = standardized coefficient; Pseudo *R* ^2^ values were calculated using the formula provided by Snijders and Bosker (2012); Age, gender, and social desirability are added as covariates at the between‐person level; Significant coefficients are bolded.

### Robustness Check

To further examine the robustness of findings on the relationships between prosocial cooking and well‐being in Study 2, we included self‐oriented cooking as an additional between‐person predictor of well‐being. The correlations between self‐oriented cooking and well‐being were small (|r| = 0.004–0.096) and comparable in magnitude to those of prosocial cooking (|r| = 0.06–0.14), indicating that both behaviors have similarly small baseline predictive strength. This supports the use of self‐oriented cooking as an appropriate robustness check to distinguish effects driven by prosocial motivation from those reflecting general cooking behavior. The pattern of results remained consistent: At the within‐person level, prosocial cooking continued to significantly predict greater same‐session positive affect, β = .064, 95% CI [0.027, 0.097], and higher momentary happiness, β = .046, 95% CI [0.009, 0.083]. At the between‐person level, prosocial cooking was not significantly associated with any well‐being outcomes (all |β| < .123, 95% CIs included zero). These findings provide further support for the robustness of the within‐person link between prosocial cooking and well‐being.

## STUDY 3: EMA OF PROSOCIAL COOKING, WELL‐BEING, AND PERSONALITY IN A COMMUNITY SAMPLE

Study 2 adopted EMA to examine within‐ and between‐person associations between prosocial cooking and well‐being among college students. However, its design had notable limitations: The sample was restricted to a narrow age range with limited opportunities for cooking (mean and between‐person variance of prosocial cooking were 0.08 and 0.03, respectively), and only three well‐being indicators were assessed. To address these issues, Study 3 recruited a larger and more diverse community sample, extended the EMA period to 14 days, and broadened the well‐being assessment to include self‐esteem and vitality (Hui & Kogan, 2018). We also explored whether personalities moderated the within‐person associations between prosocial cooking and well‐being.

## METHOD

### Participants and Procedure

A priori power analysis using Monte Carlo simulations (Lafit et al., 2021), based on the effect sizes from previous diary or EMA studies (Armstrong‐Carter & Telzer, 2021; Hui & Kogan, 2018), indicated that at least 280 participants were required to achieve .90 power for detecting small lagged effects (β = .14). We recruited a community sample by posting targeted, paid advertisements on major social media platforms (Facebook and Instagram), specifically directed at Hong Kong Chinese residents aged 18 and above. A total of 307 individuals enrolled. After excluding those with insufficient data (<3 EMA entries), the final sample comprised 295 adults (*M*_age_ = 37.31; *SD*_age_ = 12.29; 78.6% women; See Table 1 for detailed demographic information). Participants completed a baseline survey and three brief EMA surveys daily for 14 consecutive days, using the same app and procedures as in Study 2. In particular, participants completed the momentary measures in the same order as in Study 2—momentary prosocial cooking followed by momentary affect and happiness—with two additional measures (momentary self‐esteem and vitality) added in Study 3. This protocol yielded a total of 9,707 EMA entries, with participants responding to an average of 32.91 entries (*SD* = 10.16) out of 42 possible entries over two weeks (compliance rate = 78.3%).

## MEASURES

We used the same scales as in Study 2 to measure self‐oriented cooking, social desirability (ω = .51), momentary prosocial cooking, momentary positive and negative affect, and momentary subjective happiness. Details of additional measures are provided below, and their mean scores were calculated for analysis. Reliabilities for EMA scales are presented in Table 7.

**TABLE 7:** Descriptive Statistics and Inter‐correlations among the Main Study Variables in Study 3.

Variables	*M*	Variance		ICC	ω		1	2	3	4	5	6
		Within	Between		Within	Between						
1. Prosocial cooking	0.20	0.17	0.05	.22	‐‐	‐‐	‐‐	.21***	−.07	.15**	.08	.14*
2. Positive affect	4.17	0.73	1.29	.64	.90	.99	.07***	‐‐	−.57***	.87***	.75***	.83***
3. Negative affect	2.37	0.66	1.15	.64	.83	.97	−.04**	−.49***	‐‐	−.63***	−.69***	−.40***
4. Subjective happiness	4.23	0.48	1.58	.77	.76	.97	.06***	.58***	−.37***	‐‐	.75***	.64***
5. Self‐esteem	2.93	0.16	0.26	.62	.74	.98	.06***	.34***	−.35***	.34***	‐‐	.73***
6. Vitality	3.54	0.71	1.42	.67	.85	.96	.06***	.57***	−.34***	.30***	.44***	‐‐

*Note:* Descriptive statistics and inter‐correlations are based on the aggregate observed mean for each person. *M* = mean; ICC = intraclass correlation. The ω column presents the omega reliability coefficients for each variable. Prosocial cooking has only one item, so reliability is not applicable. Within‐level correlations are placed on the lower left and between‐level correlations are placed on the upper right.

* *p* < .05;

** *p* < .01;

*** *p* < .001.

### Big Five Personalities

Personality was assessed at baseline with the 20‐item Mini‐IPIP (Donnellan et al., 2006), comprising five subscales: extraversion (e.g., “I am the life of the party”; ω = .67), agreeableness (e.g., “I sympathize with others' feelings”; ω = .76), conscientiousness (e.g., “I get chores done right away”; ω = .56), neuroticism (e.g., “I have frequent mood swings”; ω = .82), and intellect/imagination (e.g., “I have a vivid imagination”; ω = .80). Items were rated from 1 (*very inaccurate*) to 5 (*very accurate*).

### Momentary Self‐esteem

Momentary self‐esteem was assessed with two items from the Rosenberg self‐Esteem Scale (Rosenberg, 1965), adapted for momentary reporting (i.e., “In the past couple of hours, I was satisfied with myself” and “In the past couple of hours, I thought I was a person of worth”) (Hui & Kogan, 2018; Weinstein & Ryan, 2010). Responses were rated from 1 (*strongly disagree*) to 4 (*strongly agree*).

### Momentary Vitality

Momentary vitality was measured with three representative items from the Subjective Vitality Scale (Ryan & Frederick, 1997), as adapted in previous studies (Hui & Kogan, 2018; Weinstein & Ryan, 2010). A sample item is, “In the past couple of hours, I felt alive and vital,” rated from 1 (*not at all true*) to 7 (*very true*).

## RESULTS

The hypotheses, methods, and most analyses for Study 3 were preregistered (https://osf.io/xqhfp/?view_only=fa123614fd634dce9530286bba4c9256), with the exception of robustness checks and cross‐level interaction analyses, which should be considered exploratory.

### Preliminary Analyses

Descriptive statistics, ICCs, and correlations are presented in Table 7. ICCs for the main study variables ranged from .22 to .77, indicating substantial variance at within‐ and between‐person levels, justifying the use of multilevel modeling.

### Multilevel Analyses

Following Study 2's analytic approach, we conducted multilevel analyses to examine within‐ and between‐person associations between prosocial cooking and well‐being, controlling for age, gender, and social desirability (see Table 6 for full results). Analyses revealed that, at the within‐person level, greater momentary prosocial cooking was significantly associated with higher momentary positive affect (β = .078, 95% CI [.057, .100]), subjective happiness (β = .061, 95% CI [.036, .081]), self‐esteem (β = .066, 95% CI [.042, .092]), and vitality (β = .056, 95% CI [.036, .078]), as well as lower negative affect (β = −.041, 95% CI [−.068, −.016]). In other words, participants reported greater well‐being after they spent more time cooking for others. At the between‐person level, participants who, on average, engaged in more prosocial cooking across the study reported higher overall positive affect (β = .168, 95% CI [.042, .273]), but no significant differences in other well‐being indicators (all|β| < .103, 95% CIs included zero).

### Robustness Check

To further establish the specificity of our findings on the relationships between prosocial cooking and well‐being in Study 3, we included self‐oriented cooking as a between‐person predictor of well‐being. Self‐oriented cooking showed small correlations with well‐being (|r| = 0.003–0.037) that were comparable in magnitude to those of prosocial cooking (|r| = 0.07–0.21). This indicates that both behaviors exhibited similarly small baseline predictive strength, supporting the use of self‐oriented cooking as a robustness check in this study. At the within‐person level, the results remained essentially unchanged: Positive associations with positive affect (β = .076, 95% CI [.052, .107]), subjective happiness (β = .059, 95% CI [.037, .091]), self‐esteem (β = .065, 95% CI [.042, .090]), and vitality (β = .057, 95% CI [.036, .081]), as well as a negative association with negative affect (β = −.040, 95% CI [−.060, −.016]). At the between‐person level, prosocial cooking remained a significant predictor of positive affect (β = .176, 95% CI [.052, .308]), and the results for other outcomes were non‐significant. These findings indicated that the within‐person effects were robust, even when accounting for the frequency of self‐oriented cooking.

### Moderating Effect of Personality

We tested cross‐level interactions with each grand‐mean‐centered personality (level 2) as a moderator of the within‐person association between prosocial cooking and well‐being, resulting in five separate moderation models. We found that extraversion and neuroticism showed significant moderating effects. To account for shared variance, both traits were included in the same model; only extraversion significantly moderated the within‐person relationships between prosocial cooking and positive affect (β = −.668, 95% CI [−.959, −.173]) and self‐esteem (β = −.238, 95% CI [−.443, −.038]).

Simple slopes were probed at ±1 *SD* from the mean of extraversion, restricted to significant moderation effects for parsimony. We found that, for positive affect and self‐esteem, both lower (−1 *SD*) and the higher (+1 *SD*) extraversion slopes were significant and positive (see Table 8). These results indicate that more introverted individuals experienced greater momentary increases in positive affect and self‐esteem during prosocial cooking episodes than more extraverted participants.

**TABLE 8:** Simple Slopes of Prosocial Cooking Predicting Well‐being Indicators at High (+1 SD) and Low (−1 SD) Levels of Extraversion in Study 3.

	−1 *SD*		+1 *SD*	
Outcomes	b	95% CI	b	95% CI
Positive affect	0.196	[0.152, 0.238]	0.144	[0.072, 0.187]
Self‐esteem	0.089	[0.057, 0.127]	0.042	[0.010, 0.077]

Abbreviations: b = unstandardized coefficient; 95% CI = 95% credibility interval.

### Cross‐lagged Associations

Finally, we examined cross‐lagged associations by modelling prosocial cooking (well‐being) at the previous session to predict well‐being (prosocial cooking) at the current session. We found no significant effects (all |β| < .022, 95% CIs included zero).

## DISCUSSION

Across four studies—including two cross‐sectional surveys and two EMA studies—we investigated the psychological benefits of prosocial cooking. Study 1b adjusted for general prosocial behavior, while Studies 2 and 3 controlled for self‐oriented cooking, isolating effects specific to preparing food for others. Consistent evidence emerged at the momentary, within‐person level: Prosocial cooking was reliably associated with increased positive affect and subjective happiness in both EMA studies, and—in our larger, community‐based sample (Study 3)—also with higher self‐esteem, vitality, and lower negative affect. In the college sample (Study 2), the within‐person association with negative affect was not significant, though the direction matched Study 3. By contrast, trait‐level associations were modest and inconsistent across studies and outcomes—most robust for positive affect, but not for other well‐being outcomes. Thus, the psychological benefits of prosocial cooking appear clearest in real‐time, situational contexts, with weaker and less reliable effects at trait‐level. Furthermore, momentary benefits were moderated by extraversion: Introverted individuals experienced greater boosts in positive affect and self‐esteem during episodes compared to their more extraverted peers. These effects were immediate and context‐dependent, with no evidence of sustained, lagged associations. Collectively, these findings highlight the importance of situational context and individual differences in understanding the emotional impact of everyday prosocial acts such as cooking for others.

This research introduces prosocial cooking as a distinct, everyday form of prosocial behavior with unique emotional dynamics. By examining the impact of cooking for others—a routine yet often‐overlooked act—we contribute to a broader understanding of how ordinary prosocial actions can shape psychological well‐being in real time (Hui & Kogan, 2018; Snippe et al., 2018; Weinstein & Ryan, 2010, Study 1). Unlike most previous research that focuses primarily on between‐person differences or on more structured, effortful forms of prosociality (for a review, see Hui et al., 2020), our studies are, to our knowledge, the first to employ EMA to capture prosocial cooking as it naturally unfolds in daily life. This intensive, within‐person approach reveals that the well‐being benefits of prosocial cooking are immediate and context‐specific, rather than stable or cumulative across individuals, thereby highlighting effects that may be masked in traditional, between‐person research designs.

Our research extends the positive‐activity model (Lyubomirsky & Layous, 2013) by demonstrating that frequent, socially embedded acts of kindness—like cooking for others—primarily confer immediate, within‐person boosts in well‐being. While our EMA studies found robust momentary benefits, we did not observe consistent trait‐level well‐being differences among individuals who engaged more frequently in prosocial cooking, aside from some evidence for higher positive affect in Study 3. This highlights that, although theory often emphasizes cumulative effects, the psychological impact of everyday prosociality may be more immediate and context‐dependent. It remains possible that frequent prosocial cooking could, over time, foster lasting improvements in well‐being—a question for future longitudinal research.

Importantly, we found that extraversion moderated the momentary well‐being benefits of prosocial cooking, supported the person‐activity fit proposed by the positive‐activity model (see Senf & Liau, 2013, for prior moderation work). Specifically, introverted individuals reported greater gains—contrasting with Snippe et al. (2018), who found no moderation by extraversion for daily prosocial behavior but did observe a role for neuroticism. This discrepancy likely reflects differences in the type and context of prosocial behavior examined. Prosocial cooking is a structured, low‐arousal, and often private act routinely embedded in daily life—features that may align particularly well with introverts' preferences for predictable and manageable interactions. In contrast, the more spontaneous and varied prosocial acts assessed by Snippe et al. may not offer the same person‐activity fit for introverted individuals. Together, these findings underscore that the psychological benefits of prosocial actions depend on the specific features of the activity and their alignment with individual personality traits (Lyubomirsky & Layous, 2013). Relatedly, self‐determination theory (SDT; Ryan & Deci, 2000) offers a complementary perspective for understanding when prosocial cooking may be most emotionally rewarding. SDT posits that well‐being is enhanced when individuals' basic psychological needs for autonomy, relatedness, and competence are satisfied. Consistent with this framework, prior research shows that everyday prosocial actions are most emotionally rewarding when they are enacted autonomously, support a sense of competence, and strengthen relatedness (Aknin, Dunn, et al., 2013; Hui & Kogan, 2018; Weinstein & Ryan, 2010). From this perspective, prosocial cooking may be especially beneficial when it is enacted voluntarily rather than out of obligation (supporting autonomy), when it involves preparing food with or for others (supporting relatedness), and when it allows individuals to feel effective in helping someone in need (supporting competence). Although these motivational processes were not directly measured in the present studies, this framework helps clarify the types of social and motivational conditions under which prosocial cooking is likely to be most strongly associated with well‐being and provide important directions for future research.

These findings have practical implications for promoting well‐being in both clinical and everyday settings. Encouraging routine, manageable acts of kindness (Aked & Thompson, 2011)—such as preparing food for others—may offer a feasible strategy to enhance daily well‐being, particularly for those less suited to socially demanding prosocial roles. In the wake of the COVID‐19 pandemic, shifts in social and domestic routines have made home meal preparation more common, and cooking has been linked to positive psychosocial outcomes (Farmer et al., 2018). Our results suggest that prosocial cooking, in particular, may serve as an accessible entry point for fostering positive affect and social connection within households and communities.

Despite its contributions, this research has several limitations. First, we observed an unexpected positive association between prosocial cooking and negative affect at the between‐person level in Study 1b. This may reflect measurement constraints—such as items inadvertently capturing stress or pressure of frequent meal preparation—or the affective complexity of prosocial cooking, which can involve obligation, performance anxiety, or fatigue (Dahl et al., 2023; Otufale & Lasisi, 2024). Future work should disentangle these potential costs and benefits, especially the possibility of ambivalence or burden when helping becomes routine. Second, our Prosocial Cooking Scale was developed and validated in Hong Kong. Although Study 3 broadened our demographic scope, our samples may not represent all cultural or demographic groups. Because practices and meanings of “cooking and providing food for others” can differ cross‐culturally, replication and validation in diverse contexts are needed to establish generalizability. Third, the Blood and Organ Donation sub‐scale showed modest internal reliability (ω = .43), which may reflect conceptual differences between blood and organ donation. Blood donation is relatively common and entails lower personal cost, whereas organ donation typically involves greater personal sacrifice (e.g., irreversible bodily donation, more substantial medical and ethical implications). We also examined the item‐total correlations and found no negative values, suggesting that the subscale is generally aligned with the broader prosociality domain. Importantly, our analyses relied on the overall prosocial behavior composite score rather than this subscale alone, and the reliability of the overall scale in our study was 0.77, comparable to that reported in the original validation study (0.78; Cheng et al., 2017). Therefore, the modest ω of this subscale would not be expected to substantially affect the results. Nevertheless, its lower reliability warrants attention.

Furthermore, as all studies were correlational, future research should adopt experimental or intervention designs to establish causality. For instance, a randomized controlled trial could instruct participants to engage in prosocial cooking (e.g., preparing meals for others) and compare their well‐being outcomes with those in a neutral or self‐focused cooking condition. Moreover, the observed effect sizes were relatively small (|β|s = .04–.24). However, even small effects can accumulate to produce meaningful long‐term outcomes (Funder & Ozer, 2019). Future studies could further explore the lasting impact of prosocial cooking on well‐being.

In addition, we did not assess the specific recipients of prosocial cooking. While most participants likely cooked for family or friends, people in many communities (e.g., Food Recovery Network, DC Central Kitchen, Sikh *langar*) also cook for strangers or those in need. The well‐being impact of prosocial cooking may vary by recipient, and future studies should examine this factor. Notably, we did not control for the effects of social contact and meal sharing to isolate the unique contribution of prosocial cooking. Previous studies have revealed the positive association between sharing meals with others and well‐being (De Neve et al., 2025; Folk & Dunn, 2025). Therefore, the positive relationship between prosocial cooking and well‐being observed in the present study may partly reflect the benefits of social contact involved in shared meals. Future research should further examine how social contact and meal sharing contribute to, or moderate, the relationship between prosocial cooking and well‐being.

In summary, our findings illuminate the unique psychological benefits of prosocial cooking—an everyday, often‐overlooked act of kindness. Although such actions may not produce enduring trait‐level changes in well‐being, they reliably provide meaningful boosts to daily emotional experience. These results underscore the value of simple, accessible acts of kindness for enhancing well‐being in everyday life.

## CONFLICT OF INTEREST STATEMENT

No potential conflict of interest was reported by the author(s).

## ETHICS STATEMENT

All studies were approved by the ethics committee of the first author's affiliated university.

## Data Availability

All study materials, anonymized data, and analysis scripts and output are publicly available (https://osf.io/brmtx/?view_only=daf77b188065491596c19c35eeae8ff2).
